# Endotoxin Neutralization as a Biomonitor for Inflammatory Bowel Disease

**DOI:** 10.1371/journal.pone.0067736

**Published:** 2013-06-24

**Authors:** Keith Champion, Laura Chiu, John Ferbas, Michael Pepe

**Affiliations:** 1 BioDtech, Inc., Birmingham, Alabama, United States of America; 2 Department of Clinical Immunology, Amgen, Inc., Thousand Oaks, California, United States of America; New York University, United States of America

## Abstract

Gram-negative bacterial endotoxin is a potent immunostimulant implicated in the development and/or progression of a variety of diseases. The mammalian immune system has both innate and adaptive immune responses to neutralize endotoxin. In this study, a system was developed to monitor bacterial exposure by measuring the extent and nature of endotoxin neutralization in plasma. In control patients, females had higher levels of endotoxin neutralization than males, mirroring clinical outcomes from bacterial infection and sepsis. In addition to the total amount of neutralization, we used inactivation techniques to elucidate the nature of this activity and develop a system to compare early and late immune responses. Using this method to monitor patients with inflammatory bowel disease, we found a more robust total response that relies more on long-term, adaptive components of the immune system and less on early, innate components. Our results indicate that endotoxin neutralization is a valuable method to discern inflammatory bowel disease patients from a control population. Additionally, the nature of neutralization may be valuable in monitoring disease severity and/or the role of medication.

## Introduction

Mammalian survival is dependent on a rapid system to neutralize the potent immunostimulatory effects of Gram-negative bacterial endotoxin, which is a lipopolysaccharide found on the bacterial membrane. This multifaceted system includes several cell types expressing toll-4 receptors, degradation enzymes and binding proteins. An acute exposure to endotoxin can result in life-threatening sepsis while chronic exposure has been implicated in several diverse disease states involving the gastrointestinal, nervous, metabolic, vascular, pulmonary and immune systems [1–6]. A standard approach to monitor the potential response to this exposure is nonexistent. This is of particular concern since there are multiple organ and tissue sites in the human body where there is a constant exposure to bacteria. In this report, we describe a new methodology which uses the ability of an individual to neutralize endotoxin to gauge bacterial exposure.

There are several significant barriers to accurate endotoxin detection in blood products. Enzymes, such as alkaline phosphatase and acyloxyacyl hydrolase, provide an innate defense against endotoxin by altering the molecule to eliminate pyrogenicity [7,8]. Immunoglobulins are the most potent defense against endotoxin and occur in two waves. Early after exposure, “natural antibodies” form an important component of the innate response. Natural antibodies are IgM molecules with broad specificity against pathogen-associated molecular patterns (PAMPs) [9]. Later in the infection, IgG are produced specifically against epitopes on endotoxin. IgG specific to core endotoxin has been shown to be extremely important in the long-term defense against bacterial infection and correlate well with clinical outcome [10]. Lastly, non-specific protein binding can neutralize circulating endotoxin. Examples of these include hemoglobin, lactoferrin and lipoproteins. These anti-endotoxin mechanisms make detection difficult and circulating endotoxin a poor indicator of infection.

Inflammatory bowel disease (IBD) is the chronic inflammation of the gastrointestinal tract that can result in abdominal pain, diarrhea and malnutrition. Crohn’s Disease (CD) and ulcerative colitis (UC) are the two major types of IBD, comprising almost all cases. The location and depth of inflammation differentiate CD from UC. In CD, the inflammation is focused on the terminal ileum but can spread to any region of the digestive tract. Inflammation in CD is not restricted to the mucosal lining and thus can invade into deep tissues of the bowel wall, even resulting in fissures, strictures and abscesses. In UC, inflammation is restricted to the mucosal lining of the colon. Therefore, CD results in deep, patchy regions of inflammation separated by healthy tissue while UC causes superficial inflammation over large, continuous regions. Both types of IBD receive a similar regimen of antibiotics, immunosuppressives and anti-inflammatory drugs, though UC relies more on anti-inflammatory drugs and less on antibiotics. Inflammation associated with IBD causes a disruption in the natural intestinal barrier leading to increased permeability. Elevated intestinal permeability has been directly demonstrated in both CD and UC, with both significantly higher than controls and CD higher than UC. This leakiness leads to issues such as diarrhea as well as significant increases in bacterial antigen exposure in the bloodstream [11–14]. It has been reported that circulating endotoxin is elevated in IBD patients and correlates with disease severity. It was also shown that the level of circulating endotoxin was slightly higher in CD than in UC, reflecting the difference in permeability [1,15]. We hypothesize that immune components against bacterial antigens are a more valuable target than circulating endotoxin. Supporting this have been numerous reports showing an increase in both IgG and IgA targeted to the core lipid A region of endotoxin in IBD. As with intestinal permeability and circulating endotoxin, these are higher in CD than in UC [1,13,16–18]. Also of importance in this work is the observation that the levels of immunoglobulins are low in patients with acute infections, such as septicemia, and only increase in patients with chronic and recurring disease, such as CD, UC or recurrent urinary tract infections (UTI) [17,19].

We believe that measuring endotoxin neutralization is an efficient and simple way to monitor immune status. In this report we use endotoxin neutralization as a gauge of the immune reaction against Gram-negative bacteria. This involves adding a known amount of endotoxin to citrated plasma samples and determining the level of endotoxin inactivated by the immune system after a defined amount of time. Additionally, we use heat and acid treatments to remove specific components of the immune response to differentiate between early and late immune reactions. In agreement with previous reports, our results indicate that females have a more robust reaction to Gram-negative infection than males. Based on the effect of age and the patterns of individual components of the immune response, we believe this is a result of elevated bacterial exposure. Extending this method to patients with IBD indicates significant differences in both the extent and nature of endotoxin neutralization. This includes a transition from early, innate immune mechanisms to adaptive measures during chronic infection. We believe that this method will be valuable in discriminating disease from control patients as well as serving as an indicator of disease severity.

## Materials and Methods

### *Ethics statement*

All plasma samples were purchased from a collection agency (www.bioreclamation.com) that collects at donor centers licensed and inspected by the FDA. The samples are from healthy, consenting and paid adult human donors. Bioreclamation maintains records of written consent. All collection and recording are under the supervision of their internal review board. Samples obtained from Bioreclamation were handled under GLP conditions in a laboratory with regular safety audits.

### *Control patients*

Control patients were assigned using a combination of a medical examination and a questionnaire. All patients underwent a brief medical evaluation requiring certain standard values for parameters such as hematocrit, blood pressure, temperature, pulse rate and weight. In addition, a cursory examination was performed to rule out obvious medical issues as well as risky behavior. The completion of the questionnaire used by the FDA to screen for blood transfusions (FDA AABB Full-Length Donor History Questionnaire) was also required. This established that the patient was not experiencing any chronic or acute pain, sore throat, major illness or drug abuse. The questionnaire also included extensive background information detailing surgery, hemophilia, sexual history and STDs (including HIV/AIDS), organ transplant, pregnancy, cancer, heart disease and menopause.

### *Preparation of crude endotoxin stock*

In all experiments a crude endotoxin stock was used instead of a purified source to more closely represent a natural infection. A frozen stock of *Salmonella enterica serovar* Typhimurium *LT2* (ATCC19585) was streaked on LB plates and grown in a 37^°^C incubator. Single colonies were transferred to sterile tubes containing 3 ml LB and incubated in a shaking 37^°^C water bath until stationary phase. The cultures were heat-lysed in a 95^°^C water bath for 10 minutes, transferred to microcentrifuge tubes and spun at 13,000 x g for 5 minutes to remove cell debris. The supernatants were removed and the heat/centrifugation steps were repeated two additional times. Removal of viable bacterial cells was verified by the absence of growth in LB broth. The stock concentration was measured using the Lonza (Basal, Switzerland) PyroGene® assay and stored at 4^°^C. The stability of the stock solution was verified at least weekly during the course of this project.

### *Endotoxin neutralization experiment*

Aliquots of 90 µl citrated human plasma were mixed with 10 µl endotoxin-free water. 72 µl of this solution was mixed with 8 µl 1000 EU/ml *Salmonella enterica serovar* Typhimurium LT2 crude endotoxin stock and incubated at room temperature for 120 minutes. Samples were then heat-inactivated in a 60^°^C water bath for 30 minutes. For determination of Heat-Sensitive Endotoxin Neutralization (HSEN), the samples were heat-inactivated in a 60^°^C water bath for 30 minutes prior to addition of endotoxin. For the determination of Acid-Sensitive Endotoxin Neutralization (ASEN), a solution of 1 M hydrochloric acid was added instead of water.

### *Endotoxin detection*

Samples from the endotoxin neutralization experiment protocol were treated with the BioDtech™, Inc. (Birmingham, AL) ESP™ treatment kit to remove the masking effects of blood plasma. This was done according to manufacturer’s specifications. Endotoxin was measured using the Lonza (Basal, Switzerland) PyroGene® assay according to manufacturer’s specifications. All tests were performed in triplicate both with and without positive product controls (PPC) with multiple sets of standards, blanks and controls. Results are given as the average ± standard deviation of all three tests.

### *Neutralization calculation*

Total Endotoxin Neutralization (TEN), HSEN, ASEN and Heat- and Acid-Resistant Endotoxin Neutralization (HAREN) were determined by repeating the neutralization and detection experiments with the same patient sample under different treatment conditions (i.e. no treatment, heat-inactivation, acidification) and comparing them with each other and control reactions. TEN was determined by subtracting the amount of recovered endotoxin in an untreated plasma sample to the amount of endotoxin added, determined by multiple control reactions (e.g. 20 EU/ml detected from a 100 EU/ml spike results in a recovery of 20% and TEN of 80%). HSEN was determined by subtracting the amount of endotoxin recovery in the heat-treated samples from the recovery used to determine TEN (e.g. 40 EU/ml detected from a 100 EU/ml spike results in a recovery of 40% and a HSEN of 20% (40%-20% (from TEN calculations)). ASEN was determined by subtracting the amount of endotoxin recovery in the acid-treated samples from the recovery used to determine HSEN (e.g. 90 EU/ml detected from a 100 EU/ml spike results in a recovery of 90% and an ASEN of 50% (90%-40% (from HSEN calculations). HAREN was determined by subtracting the amount of endotoxin recovered from the acidified samples from the total amount of endotoxin added (e.g. 90 EU/ml detected from a 100 EU/ml spike results in recovery of 90% and a HAREN of 10%).

### *Statistics*

All experiments were performed in triplicate. Individual data points are given as the average with error bars representing the standard deviation. For HSEN, ASEN and HAREN the indicated standard deviations were determined using the equation √(SD1^2^ + SD2^2^). For each sample group the mean and standard error are indicated. Statistical significance was determined by comparing the values in each group against the control group with Student’s t-test. The n number for each group was 10 for control males, 10 for UC males, 10 for CD males, 20 for control females, 16 for UC females and 20 for CD females.

## Results

It has been well established that there are components in mammalian blood that neutralize endotoxin [20–22]. This report attempts to quantitate and characterize endotoxin neutralization to monitor immune system activation in response to Gram-negative bacterial exposure and then test that system in patients with IBD. Consistent with previous reports, we found that a purified endotoxin stock was almost completely (>99%) neutralized after incubation in plasma for 10 minutes [22]. Heating the plasma prior to endotoxin addition decreased the level of neutralization significantly, suggesting that heat-labile factors, such as IgM and enzymatic activity, were responsible for a portion, but not all of the neutralization [20]. Next, we compared this to the neutralization of a crude endotoxin lysate prepared without extraction techniques to more closely resemble a natural infection. We found that neutralization was significantly reduced, consistent with reports showing that purified endotoxin is a poor representative of natural infection [23,24].

**Figure 1:**
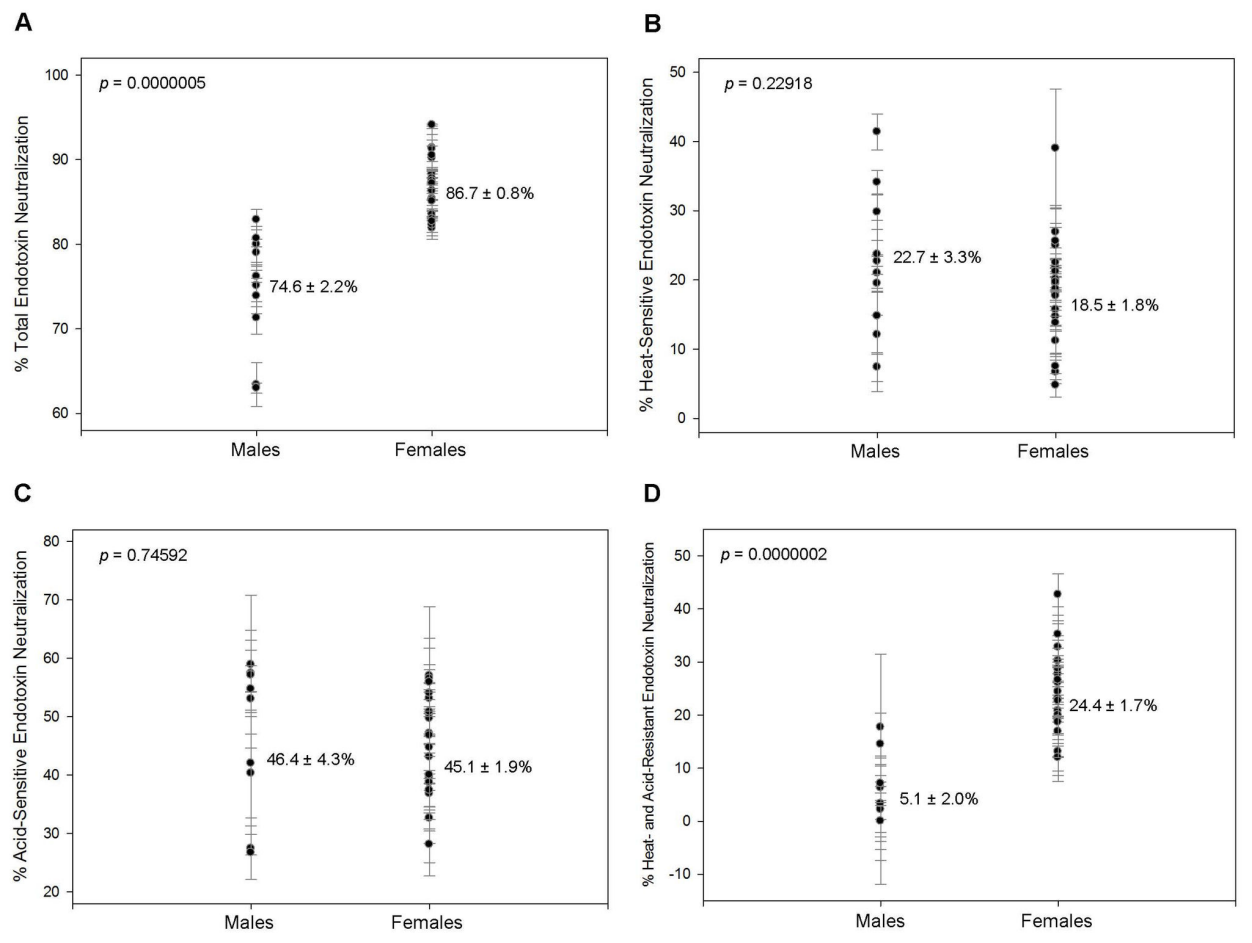
Endotoxin neutralization. Each data point with error bar represents the average and standard deviation of 3 replicates. The mean and standard error of the mean are indicated for each sample group. The *p* value indicates the statistical difference between males and females of each group as determined using Student’s t-test. (A) TEN was 74.6 ± 2.2% in males and 86.7 ± 0.8% in females. This difference was statistically significant (*p* = 0.0000005) with only 1 overlapping sample. (B) HSEN was 22.7 ± 3.3% in males and 18.5 ± 18.5% in females. (C) ASEN was 46.4 ± 4.3% in males and 45.1 ± 1.9% in females. (D) HAREN was 5.1 ± 2.0% in males and 24.4 ± 1.7% in females. This difference was statistically significant (*p* = 0.0000002) with only 2 overlapping samples.

**Figure 2:**
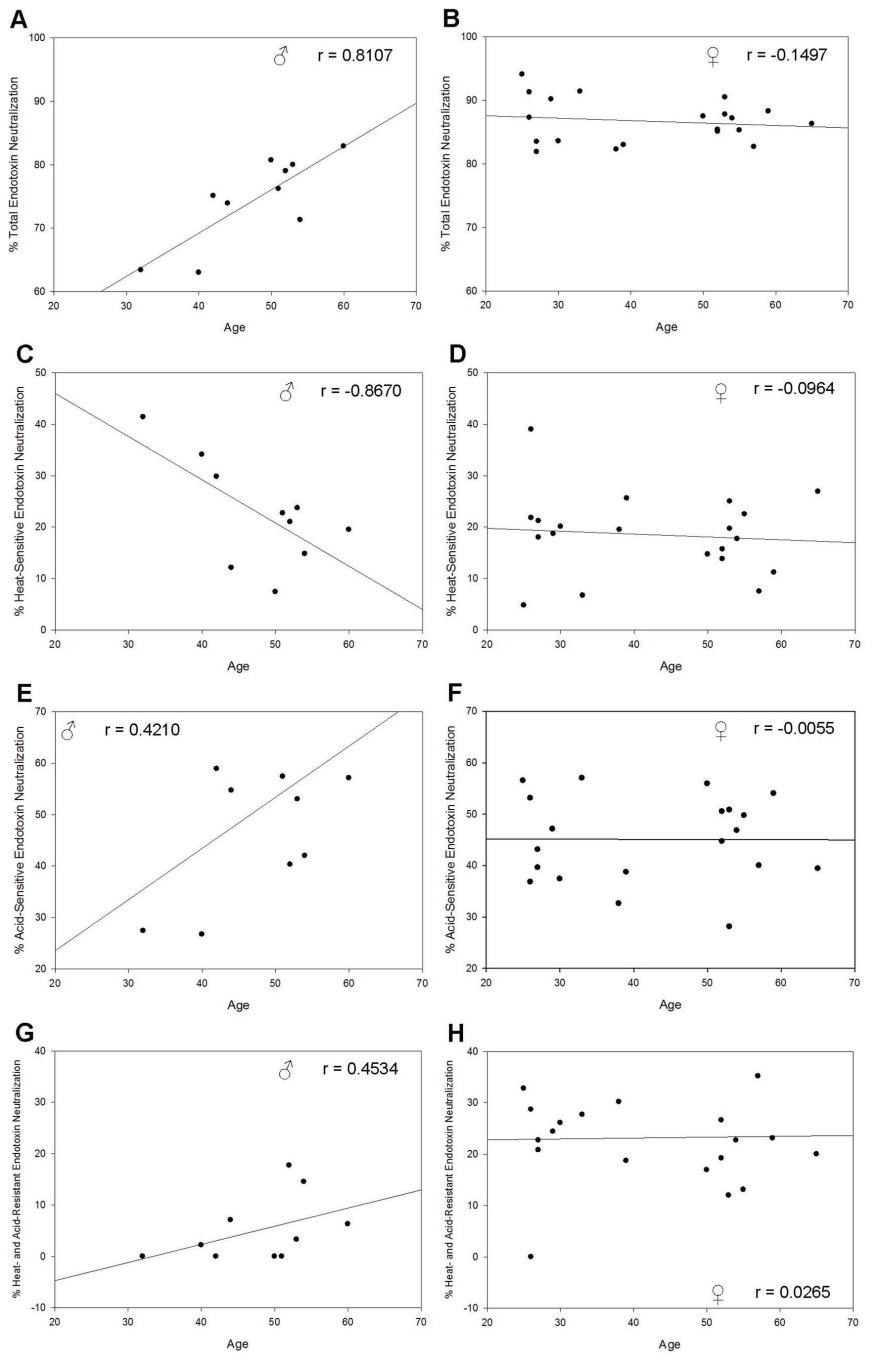
Effect of age on endotoxin neutralization. The average of 3 replicates was determined for each neutralization value and the result was plotted against patient age. A linear regression line was fit to the data and the r value was determined. For each sample set the male graph is on the left and the female is on the right. (A–B) TEN increases with age in males (*r* = 0.8107) but stays relatively constant in females (*r* = -0.1497). (C–D) HSEN decreases with age in males (*r* = -0.8670) but stays constant in females (*r* = -0.0964). (E–F) ASEN increases with age in males (*r* = 0.4210) but stays constant in females (*r* = -0.0055). (G–H) HAREN increases with age in males (*r* = 0.4534) but stays constant in females (*r* = 0.0265).

Given these results, circulating endotoxin is a poor biomonitor. However, it is possible that the level of endotoxin neutralization may indicate the level of immune system activation. To test this, we collected 30 citrated plasma samples from normal, human adults (10 male (age 32-60), 20 female (age 25-65)). The control population was defined by FDA criteria for blood transfusions to avoid patients with underlying immune activation. After assuring that the samples contained no circulating endotoxin, each sample was spiked to a final concentration of 100 EU/ml using a crude endotoxin stock solution, allowed to incubate at room temperature and measured for remaining endotoxin. The percentage of endotoxin “neutralized” was determined and defined as “total endotoxin neutralization” (TEN). The mean TEN value was significantly (p = 0.0000005) higher in females than males (Figure 1A). Males had a mean of 74.6 ± 2.2% compared to 86.7 ± 0.8% in females. Fitting a linear regression line to an age vs. TEN scatter plot gave a correlation coefficient r-value of 0.8107 in males (Figure 2A) compared to -0.1497 in females (Figure 2B). This suggests that TEN increases with age in males but remains constant in females. These results are consistent with previous reports showing that females have a more effective immune response against bacterial pathogens resulting in a better prognosis than men from sepsis and severe infection [25,26]. This is most likely due to a more vigorous immune reaction in response to higher susceptibility to infection. For example, urinary tract infections (UTI), which account for the majority of bacterial infections, are 4 times more likely in females than males with nearly 1 in 3 women having a UTI requiring antibiotic treatment by the age of 24 [27]. In agreement with this, females have significantly higher levels of IgM, the principle immunoglobulin against endotoxin [28,29]. This early, more robust expression of immunoglobulins in females helps explain the differential effects of age on TEN.

These results suggest that endotoxin neutralization is an effective and simple means to monitor immune system activation in response to Gram-negative bacterial exposure. As previously noted, endotoxin neutralization in mammalian blood relies on at least two distinct systems [20,21,30]. In the following sections the use of heat and acidification to remove components of the immune system was investigated to characterize the mechanisms of endotoxin neutralization.

Blood plasma contains many enzymes that destroy and detoxify endotoxin. It has been well established that heating plasma will remove enzymatic activity and reduce the amount of neutralization [21–23]. In addition to enzymes, IgM, the principle immunoglobulin against endotoxin that appears early after infection, is very sensitive to heat [31,32]. Alternatively, IgG, especially the antigen-binding region, is much more heat-tolerant. The heat-sensitivity of other plasma proteins varies. Therefore, we hypothesize that heat-inactivation of plasma will remove much of the early response to bacterial infection, like enzymes and IgM, with little effect on factors of the adaptive response that appear later. To determine the portion of TEN caused by heat-sensitive factors, we repeated the neutralization experiments using the same plasma samples after heat-inactivation at 65^°^C for 30 minutes. The level of endotoxin remaining in the heat-inactivated samples was then subtracted from the level of endotoxin remaining in the TEN experiments to give the level of endotoxin neutralization that can be attributed to heat-sensitive factors. This was termed “heat-sensitive endotoxin neutralization” (HSEN) and was very similar between the sexes (Figure 1B). The mean in males was 22.7 ± 3.3% compared to 18.5 ± 1.8% in females. Though the mean HSEN was similar between the sexes, the trend with age was distinct. In the male data, the r-value was -0.6699 indicating that as age increased the level of HSEN decreased (Figure 2C). As with TEN, there was no age component in females (Figure 2D).

Heat denatures many structural proteins in addition to IgM and enzymatic activity. Therefore the HSEN value does contain some protein-binding neutralization. However, there are issues with heat denaturation of many proteins, specifically immunoglobulins. IgG, especially the antigen-binding region, is resistant to heat denaturation and more sensitive to pH changes. Also, IgG heat-denaturation occurs so quickly that they often aggregate, allowing them to become active after the heat is removed [33]. The differences in heat-denaturation on IgG and IgM are so great that it is as effective as flow cytometry in distinguishing the populations [31]. Therefore, heat-inactivation reflects the loss of enzymes and some proteins, such as IgM, while acidification reflects the loss of the majority of remaining proteins, including partial denaturation of IgG. To determine the extent of TEN caused by pH-sensitive factors we repeated the neutralization experiments using the same plasma samples treated with a strong hydrochloric acid solution. In acidification of the samples, most heat-labile components would also be denatured. Therefore, the level of endotoxin remaining after incubation with acid-treated plasma was subtracted from this same level in the HSEN experiments to establish the level of neutralization caused by acid-sensitive, but not heat-sensitive, components. This was termed “acid-sensitive endotoxin neutralization” (ASEN). ASEN was similar in males and females (Figure 1C). In males, the mean ASEN was 46.4 ± 4.3% compared to 45.1 ± 1.9% in females. The r-value of the male data was 0.4210 compared with -0.0055 in females (Figure 2, E and F). This suggests a modest increase in acid-labile endotoxin neutralization with age in males but no correlation in females.

Acid treatment of plasma should denature most of the proteins and enzymes. However, some proteins may persist after treatment [21]. Specifically, it has been demonstrated that a significant amount of the secondary structure of immunoglobulins can remain even after acidification [33]. Using the values from the ASEN experiments and comparing them to the endotoxin stocks and control experiments we were able to determine the level of endotoxin neutralization that persisted after both heat and acid treatment. This portion of endotoxin neutralization was termed “heat- and acid-resistant endotoxin neutralization” (HAREN). We found that HAREN was much higher in females than in males (Figure 1D). This difference was statistically significant (p = 0.0000002). In males, the mean HAREN was 5.1 ± 2.0% compared to 24.4 ± 1.7% in females. As with TEN and ASEN, male HAREN increased with age (Figure 2G) but female HAREN remained constant (Figure 2H). Also similar to TEN, male HAREN increased with age from a very low value to a maximum value roughly equivalent to the average female HAREN. We propose that this portion of endotoxin neutralization consists of proteins with high-affinity to endotoxin and explains why the patterns are similar to TEN.

**Figure 3:**
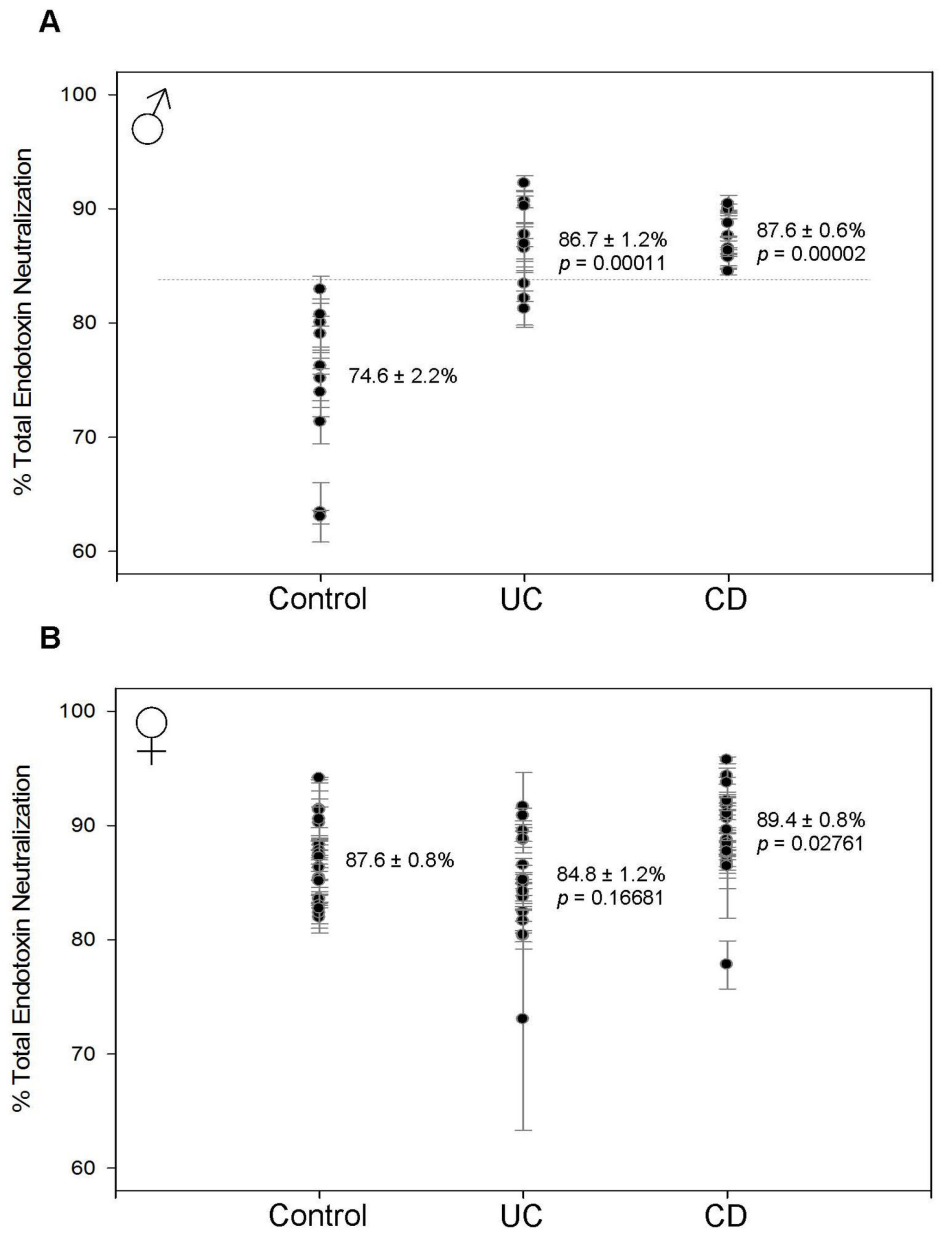
Total endotoxin neutralization (TEN). Each data point with error bar represents the average and standard deviation of 3 replicates. The mean and standard error of the mean are indicated for each sample group. The *p* value indicates the statistical difference compared with the control group as determined using Student’s t-test. (A) In males, TEN is increased from 74.6 ± 2.2% in controls to 86.7 ± 1.2% (*p* = 0.00011) in UC and 87.6 ± 0.6% (*p* = 0.00002) in CD. A line on the y-axis at 83% correctly demarcates control from UC with 95% accuracy and from CD with 100% accuracy. (B) In females, TEN is increased from 87.6 ± 0.8% in controls to 84.8 ± 1.2% (*p* = 0.16681) in UC and 89.4 ± 0.8% (*p* = 0.02761) in CD.

To test the ability to monitor Gram-negative bacterial exposure using endotoxin neutralization we used samples from patients with IBD. Given the differences between sexes, plasma samples from patients with Crohn’s Disease (CD) (10 male, 20 female) and ulcerative colitis (UC) (10 male, 16 female) were tested and compared to the same sex controls. In patients with IBD, the value of TEN was similar for both males and females with CD and UC. In males, mean TEN was significantly higher than controls for both CD (87.6 ± 0.6%, p = 0.00002) and UC (86.7 ± 1.2%, p = 0.00011) (Figure 3A). In females, TEN was slightly, but significantly, increased in CD (89.4 ± 0.8%, p = 0.02761) with no significant change in UC (84.8 ± 1.2%, p = 0.16681) (Figure 3B). TEN appears to be a valuable indicator of IBD in males. A horizontal line at a value of 83% on the y-axis correctly demarcates the control population from the CD population with 100% accuracy and from the UC population with 95% accuracy. However, given the elevated TEN value in the controls this method is not applicable in females. The observation that neutralization is higher in IBD and slightly higher in CD than UC is mirrored by similar observations with intestinal permeability, circulating endotoxin and the level of bacterial antigens bound by immunoglobulins [1,13,18].

**Figure 4:**
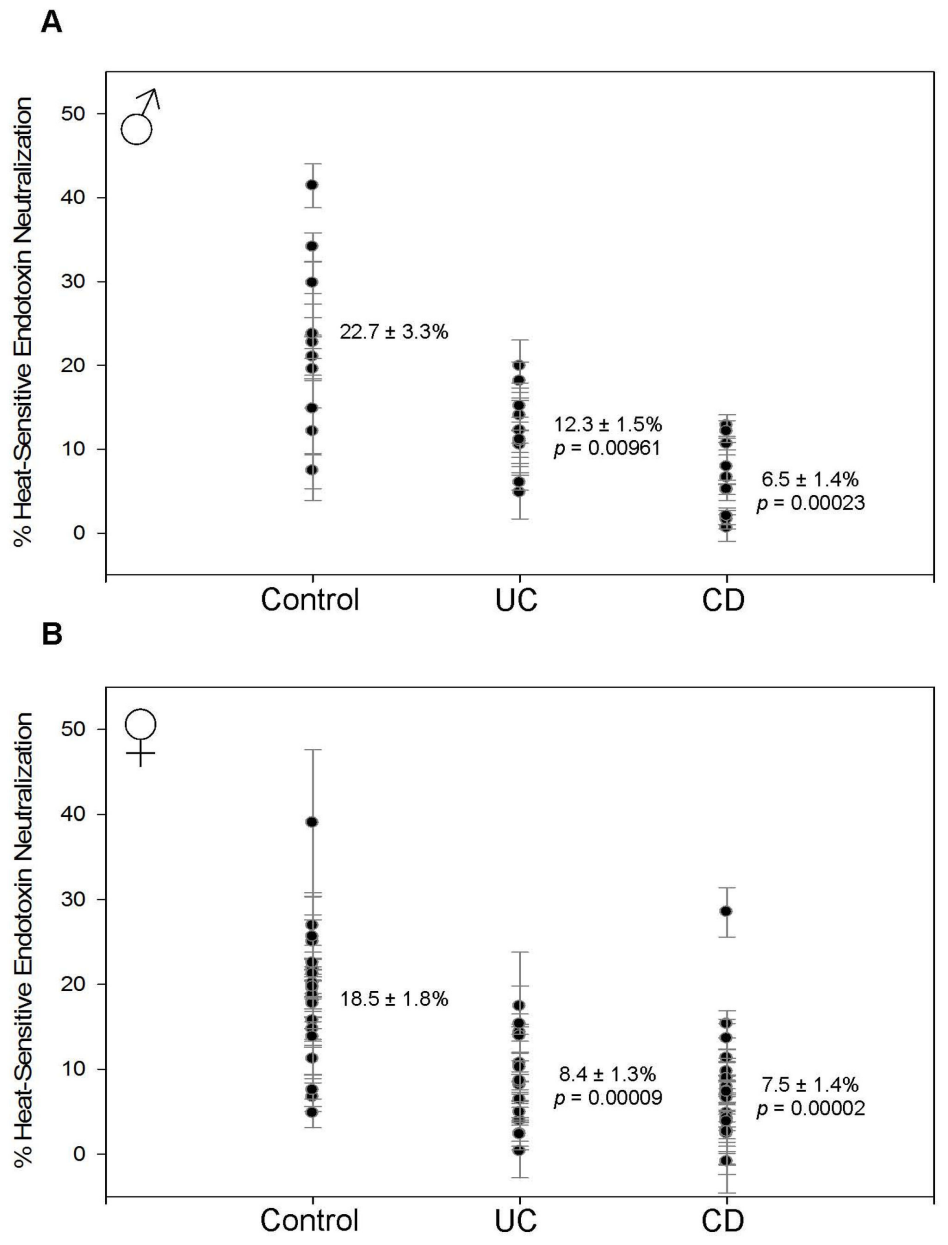
Heat-sensitive endotoxin neutralization (HSEN). Each data point with error bar represents the average and standard deviation of 3 replicates. The mean and standard error of the mean are indicated for each sample group. The *p* value indicates the statistical difference compared with the control group as determined using Student’s t-test. (A) In males, HSEN is decreased from 22.7 ± 3.3% in controls to 12.3 ± 1.5% (*p* = 0.00961) in UC and 6.5 ± 1.4% (*p* = 0.00023) in CD. (B) In females, HSEN is decreased from 18.5 ± 1.8% in controls to 8.4 ± 1.3% (*p* = 0.00009) in UC and 7.5 ± 1.4% (*p* = 0.00002) in CD.

**Figure 5:**
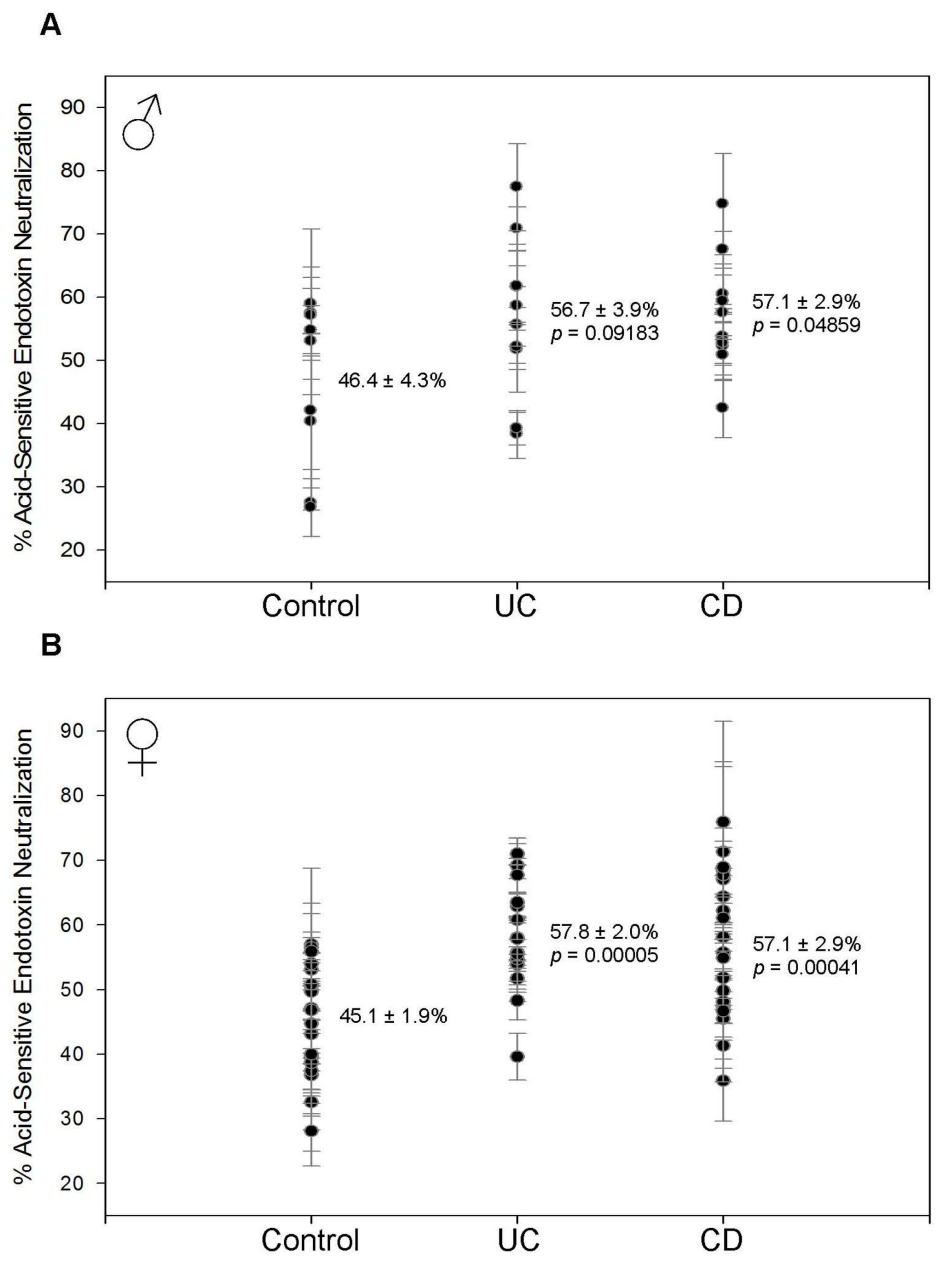
Acid-sensitive endotoxin neutralization (ASEN). Each data point with error bar represents the average and standard deviation of 3 replicates. The mean and standard error of the mean are indicated for each sample group. The *p* value indicates the statistical difference compared with the control group as determined using Student’s t-test. (A) In males, ASEN is increased from 46.4 ± 4.3% in controls to 56.7 ± 3.9% (*p* = 0.09183) in UC and 57.1 ± 2.9% (*p* = 0.04859) in CD. (B) In females, ASEN is increased from 45.1 ± 1.9% in controls to 57.8 ± 2.0% (*p* = 0.00005) in UC and 57.1 ± 2.9% (*p* = 0.00041) in CD.

Both male and female patients with IBD have decreased HSEN (Figure 4) and increased ASEN (Figure 5). This suggests that the innate response is being replaced by the adaptive response due to long-term bacterial exposure. The decrease in HSEN is between 10.1–16.2% and statistically significant in all groups. In males, HSEN measured 6.5 ± 1.4% (p = 0.00023) in CD and 12.3 ± 1.5% (p = 0.00961) in UC compared to 22.7 ± 3.3% in controls (Figure 4A). These results are very similar to females where the 18.5 ± 1.8% control value was reduced to 7.5 ± 1.4% (p = 0.00002) in CD and 8.4 ± 1.3% (p = 0.00009) in UC (Figure 4B). In each of the groups there is an increase in ASEN that is roughly equivalent to the decrease of HSEN. In males, ASEN was increased from 46.4 ± 4.3% in controls to 57.1 ± 2.9% (p = 0.04859) for CD and 56.7 ± 3.9% (p = 0.09183) for UC (Figure 5A). The female statistics are almost identical. ASEN was increased from 45.1 ± 1.9% in the controls to 57.1 ± 2.9% (p = 0.00041) in CD and 57.8 ± 2.0% (p = 0.00005) in UC (Figure 5B).

**Figure 6:**
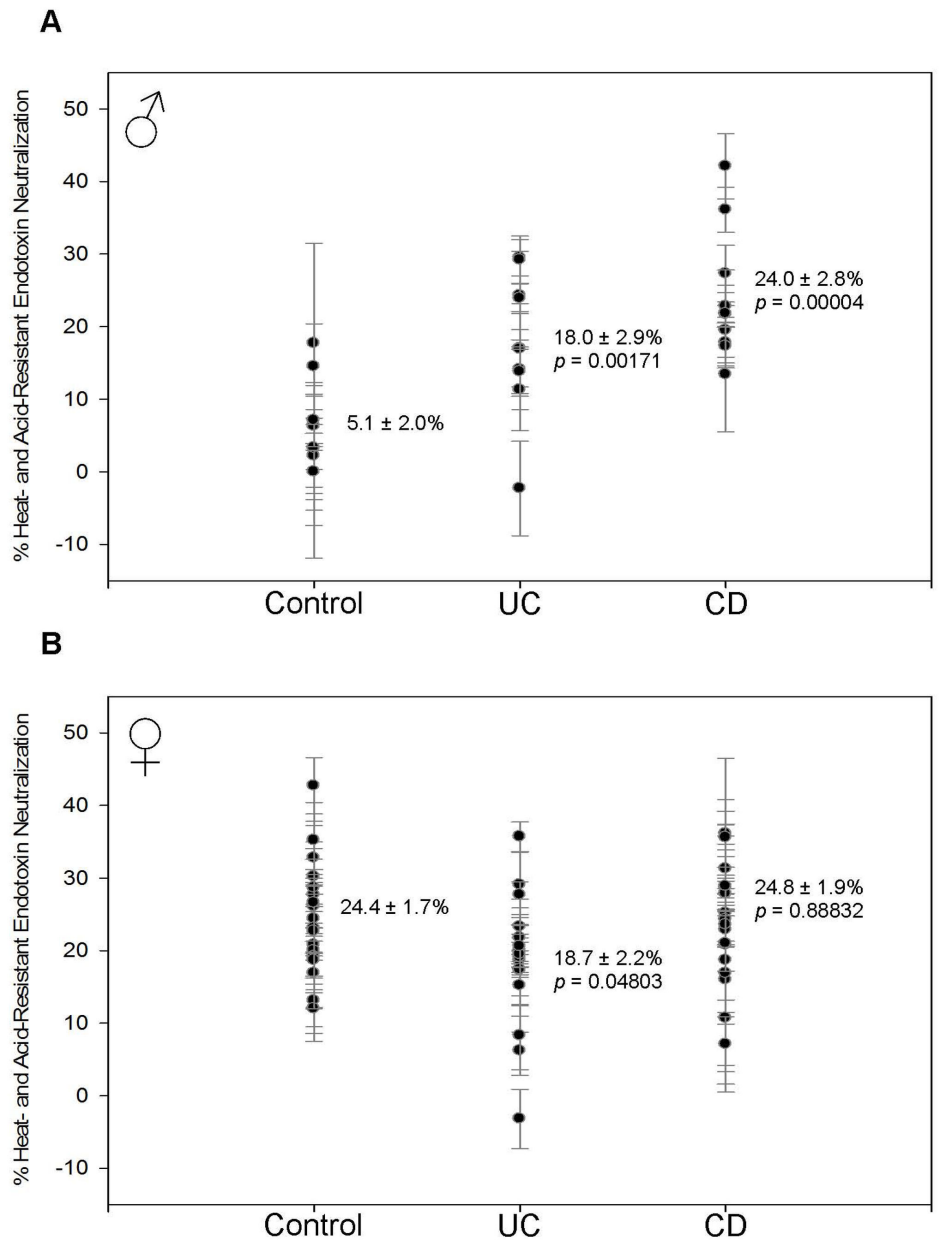
Heat- and acid-resistant endotoxin neutralization (HAREN). Each data point with error bar represents the average and standard deviation of 3 replicates. The mean and standard error of the mean are indicated for each sample group. The *p* value indicates the statistical difference compared with the control group as determined using Student’s t-test. (A) In males, HAREN is increased from 5.1 ± 2.0% in controls to 18.0 ± 2.9% (*p* = 0.00171) in UC and 24.0 ± 2.8% (*p* = 0.00004) in CD. (B) In females, HAREN is decreased from 24.4 ± 1.7% in controls to 18.7 ± 2.2% (*p* = 0.04803) in UC and unchanged at 24.8 ± 1.9% (*p* = 0.88832) in CD.

Given that the changes in HSEN and ASEN roughly cancel each other out, HAREN is responsible for the changes in TEN. In males there is a dramatic and significant increase in HAREN from 5.1 ± 2.0% in the controls to 24.0 ± 2.8% (p = 0.00004) for CD and 18.0 ± 2.9% (p = 0.00171) in UC (Figure 6A). In IBD samples, female HAREN was almost identical to males. However, since control males and females have such different HAREN, due to differences in bacterial exposure, there is little change in females where controls measure 24.4 ± 1.7% compared to 24.8 ± 1.9% (p = 0.88832) in CD and 18.7 ± 2.2% (p = 0.04803) in UC (Figure 6B).

The ultimate goal of this report was to develop a simple system to monitor bacterial exposure. TEN shows great promise in achieving this goal in males. However, by comparing the specific factors of the immune system being activated during chronic endotoxin exposure, we developed a metric that is applicable in all samples. A factor describing the ratio of immune components responsible for long-term protection to those appearing quickly after infection would be valuable for this purpose. Therefore, we developed the “endotoxin neutralization ratio” (ENR) which is defined as (ASEN + HAREN)/HSEN. In addition to the above attributes, ENR removes much of the volatility associated with age (Figure S1).

**Figure 7:**
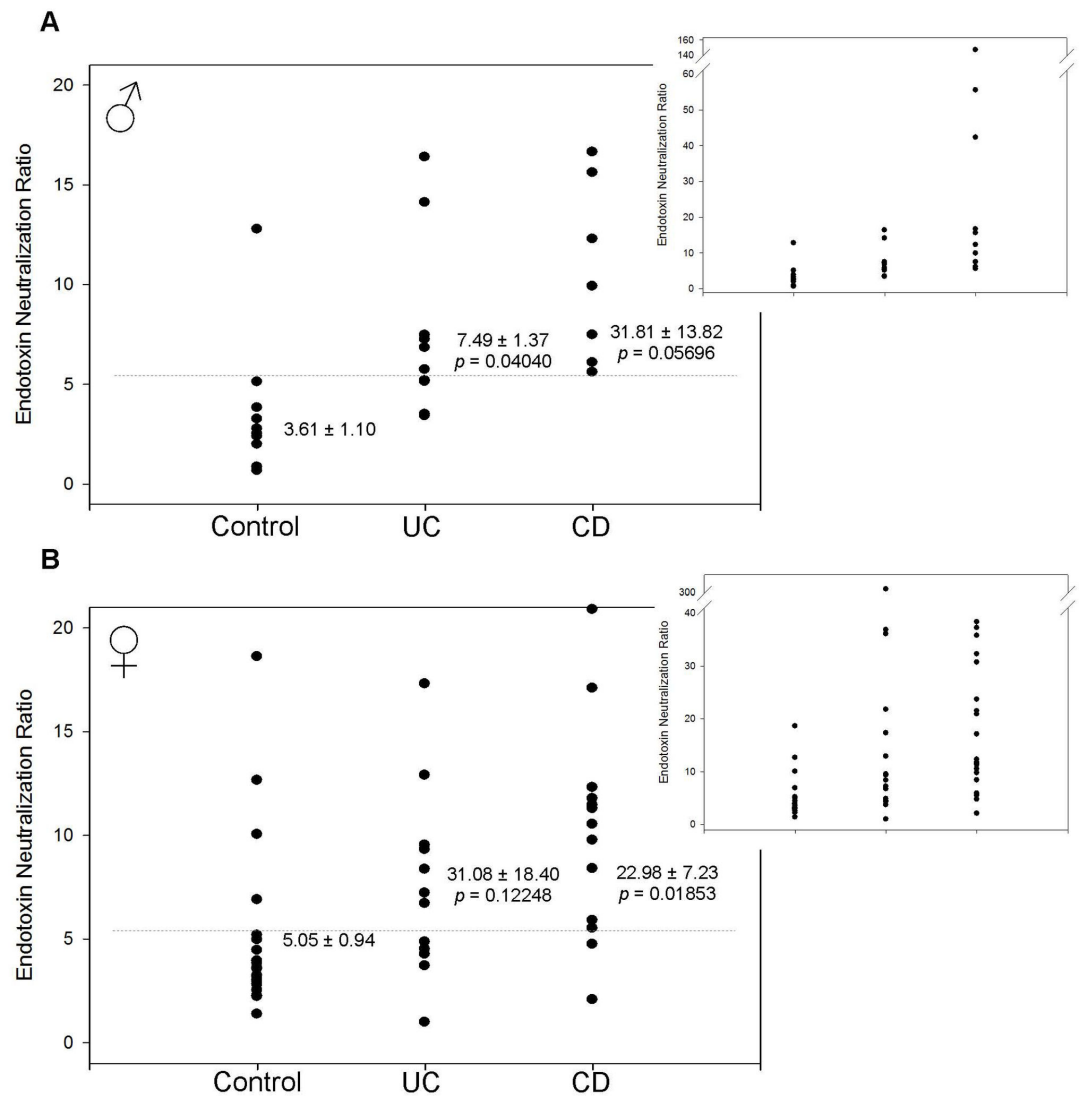
Endotoxin neutralization ratio (ENR). The ENR of each patient sample was determined with the equation ENR = (ASEN + HAREN)/HSEN using the average of 3 replicates for each test. The mean and standard error of the mean are indicated for each sample group. The *p* value indicates the statistical difference compared with the control group as determined using Student’s t-test. Each graph includes an inset with an expanded y-axis to demonstrate significantly higher ENR values for UC and CD. (A) In males, ENR is increased from 3.61 ± 1.10 in controls to 7.49 ± 1.37 (*p* = 0.04040) in UC and 31.81 ± 13.82 (*p* = 0.05696) in CD. A line of demarcation at 5.3 correctly indicates UC and CD with 85% and 95% accuracy, respectively (B) In females, ENR is increased from 5.05 ± 0.94 in controls to 31.08 ± 18.40 (*p* = 0.12248) in UC and 22.98 ± 7.23 (*p* = 0.01853) in CD. If the outlier at 304.33 in the UC population is omitted the mean becomes 12.86 ± 2.79 (*p* = 0.00564). Including the outlier, a line of demarcation at 5.3 correctly indicates UC and CD with 78% and 85% accuracy, respectively.

**Figure 8:**
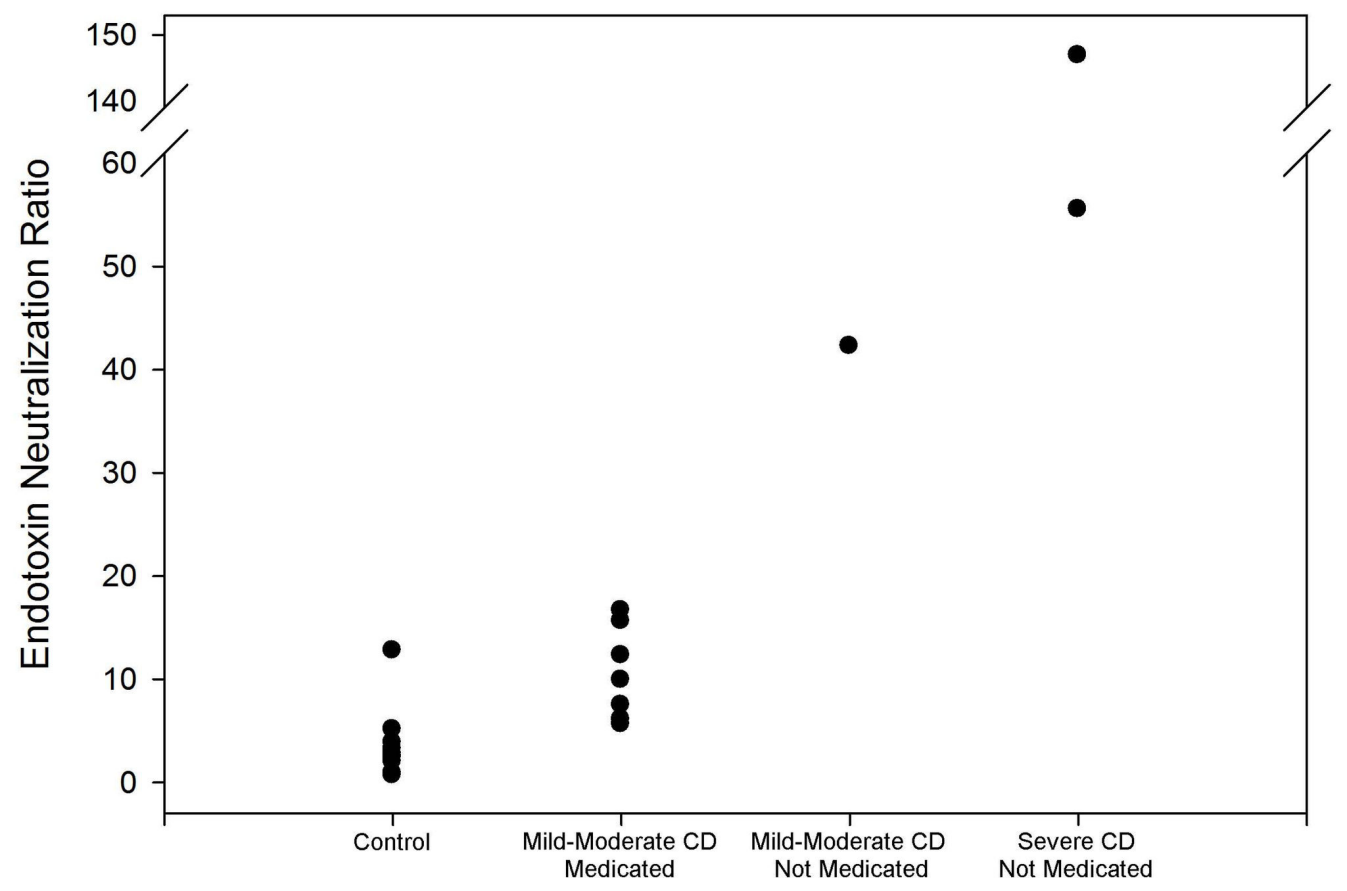
ENR as indicator of CD severity. ENR has value as an indicator of disease severity/medication in CD. In males, controls ranged from 0-13 with a mean ± SEM of 3.61 ± 1.10. In patients with mild-to-moderate CD receiving an immunomodulator and/or antibiotic this increased the range to 5-17 with a mean of 10.51 ± 1.69. The single patient with moderate CD receiving no immunomodulator or antibiotic had an ENR of 42.25. Finally, there were 2 patients with severe CD, both receiving neither drug type. These patients had the highest ENRs, 55.50 and 146.83. Age was not a factor.

Control male ENR samples clustered in an area between 0-6 with one exception at 12.76 (Figure 7A). The mean was 3.61 ± 1.10. Male CD samples were much more diverse and skewed toward higher values. There was only 1 sample in the 0-6 range with 3 more between 6–10. There was a cluster of 3 patients between 10–20 and individual points at 42.24, 55.50 and 146.83. The mean for male CD was 31.81 ± 13.82. Due to a large SEM this result was borderline statistically significant (p = 0.05696) but there was only 1 sample that overlapped the two populations. As with TEN, a line of demarcation on the y-axis at a value of 5.3 correctly distinguishes control from CD samples with 95% accuracy. Male UC samples were intermediate between control and CD. There was a cluster of 8 patients between 3–8 with the other two samples at 14.10 and 16.38 for a mean of 7.49 ± 1.37. Though this was only a slight increase it was statistically significant (p = 0.04040) and the line at 5.3 correctly segregates control from UC with an accuracy of 75%. In addition to a simple “yes or no” determination in CD, ENR correlates with disease severity and/or medication, though the sample sizes are too small to be statistically significant (Figure 8). The 7 patients with the lowest ENR values had either mild to moderate cases of CD and were prescribed either an immunomodulator or antibiotic. The patient at 42.25 had a mild case of CD but was not prescribed either drug type (though he was prescribed an anti-inflammatory). The patients at 55.50 and 146.83 both had severe cases of CD and were not prescribed an immunomodulator, antibiotic or anti-inflammatory. It was not possible to determine if severity and/or medication affected ENR in UC samples since all patients had mild to moderate symptoms and an overlap of medications. The overall value of this method for measuring disease severity naturally rests on collection of more data, which would include cases of mild-moderate CD with no medications and more cases of severe CD with and without medications. We nonetheless include this data at this time such that others may consider this approach as we expand the scope of assessments in our own laboratory.

The ENR from control females had a similar pattern to the control males (Figure 7B). Sixteen of the samples were clustered in an area below 6 with 4 additional samples spread out between 6–19. The mean value was 5.05 ± 0.94. Also similar to males, the CD samples were much more spread out with the UC samples intermediate. For CD there were 2 patients that gave values similar to the average control (2–5) and two more patients that were slightly higher (5,6). Next was a cluster of seven patients between 8–13. Even higher were two populations, one with 5 patients between 17–27 and another with 3 patients between 30–40. Lastly there was one patient with an ENR of 153.17. The mean female CD was 22.98 ± 7.23 (p = 0.01853) and a line of demarcation at 5.3 correctly segregates control from CD with an accuracy of 85%. In female UC, there were 10 samples from 3–10, a group of 5 between 12–37 and a single outlier at 304.33. Using all samples the mean was 31.08 ± 18.40 and was not significantly different (p = 0.12248) than the control population due to the outlier. If this single point is omitted the mean becomes 12.86 ± 2.79 which makes the population statistically significant (p = 0.00564), similar to the male UC data and intermediate between female control and female CD. A line at 5.3 correctly distinguishes control from UC with an accuracy of 78%. As with males, disease severity and/or medication may affect ENR in CD. Three of the highest 4 ENR values were not prescribed an immunomodulator, antibiotic or anti-inflammatory. Conversely, 15 of the lowest 17 values were prescribed at least one of these medications. As with the males, we were not able to determine a correlation between ENR and disease severity/medication in UC due to the sample set. All but one patient had mild to moderate UC and all but one patient was prescribed a similar medication regimen.

TEN is capable of distinguishing control from disease samples with 98% accuracy in males. ENR can distinguish control from disease samples with 85% accuracy in males and 82% in females and may indicate disease severity and/or the effects of medication.

## Discussion

The mammalian immune system has both innate and adaptive mechanisms to inactivate bacterial endotoxin. Human plasma contains enzymes, such as phosphatases and hydrolases, which can rapidly detoxify endotoxin [7,8]. In addition, plasma proteins, such as apolipoprotein, hemoglobin and lactoferrin neutralize endotoxin and impair detection with both LAL and cytokine expression assays [34–36]. The most prevalent neutralizing activity is due to endotoxin-specific immunoglobulins. Increases in immunoglobulins, especially IgM, correlate to survival in septic patients and have been used in IV-enrichment treatments [37,38]. Given the extent and variability of neutralizing activity in the bloodstream, circulating endotoxin is an inappropriate biomonitor. A careful examination and quantitation of endotoxin neutralization has never been fully explored. In this report, we have used this observation to establish and characterize an easy method to monitor the mammalian immune system in response to Gram-negative bacterial exposure.

There are three significant technical issues related to using endotoxin neutralization to monitor bacterial exposure. The first is the source of endotoxin. Most studies in the literature use stocks of endotoxin that have been prepared by various extractions methods that typically include chloroform, phenol, ether, acids and/or detergents. However, the use of extracted endotoxin is problematic for several reasons. Extracted endotoxin is not present in nature and therefore not encountered by the native immune system. Endotoxin in nature is complexed in cell wall structures and associated with other molecules that can affect both immunogenicity and pyrogenicity [39,40]. Extraction methods can cause dramatic reorganization of cell wall components, cause the formation of hybrid molecules and created biologically active subfractions [41,42]. Differences in protein-rich and protein-free endotoxin samples have been demonstrated as well as differences due to bacterial species, smooth or rough strains and different isolation procedures [23,24]. Because of these reasons we chose to use an endotoxin stock prepared from an in-house culture, gently heat-lysed and centrifuged to remove cell debris. In addition to endotoxin purity, bacterial species was important. Preliminary experiments showed that a crude stock of *Escherichia coli N99* (ATCC 33956), a species found in normal gut flora, was almost completely neutralized (>90%) within a few minutes. This is mostly due to specific anti-endotoxin immunoglobulins since all patients have had exposure to this species. Therefore, we chose to use endotoxin from *Salmonella enterica serovar* Typhimurium *LT2* (ATCC19585), a species not normally found in gut flora. The immune response against this non-native strain is less specific and therefore provided a basal level of neutralization in the control group from which changes in the disease population could be detected and quantitated. The second issue is the proper anticoagulant to use during blood collection. The Limulus amebocyte lysate (LAL) assay is based on the blood clotting system of the horseshoe crab and is therefore sensitive to many anticoagulants. We choose to perform these experiments using blood plasma containing sodium citrate as an anticoagulant. Initial results suggested that citrated or EDTA plasma was applicable for these experiments but heparinized plasma and serum were not. This is consistent with previous observations which suggested divalent cations play a role in endotoxin neutralization [20,21]. The last issue involves the detection assay. The LAL assay contains multiple sites for possible inhibition and enhancement. It is non-specific due to its reactivity with glucans associated with yeast and suffers from lot-to-lot variability. The enzymes involved in clot formation in LAL are homologues to those found in humans and thus human clotting proteases present in sera and plasma can cause false positives. To circumvent these potential problems we chose to use an assay that relies solely on a recombinant form of Factor C for detection. Factor C is a zymogen, which is activated by endotoxin binding and then cleaves a synthetic peptide substrate producing florescence which is quantitative. This approach allows us to investigate specificity and variability related to endotoxin detection.

It is well-established that there are differences in the male and female immune systems that result in females having a more robust reaction to bacterial infection. Females have a significantly better prognosis from sepsis than men as well as significantly lower hospital mortality and a better immune response to traumatic injury [25,43,44]. The elevated incidence of autoimmune disorders is another indicator of the vigor of the female immune system [45]. Complementary to these observations, our data show that the level of total endotoxin neutralization (TEN) is significantly higher in females than males (Figure 1A). This correlation suggests that the use of endotoxin neutralization is a good indicator of immune system activation. Internal to TEN, we attempted to further characterize neutralization. We hypothesized that early immune responses, such as enzymatic activity associated with the innate response and IgM, which appears soon after bacterial infection, could be removed by standard heat-inactivation (HSEN). Conversely, later immune responses, specifically IgG, would withstand heat but could be removed by acidification (ASEN). Lastly, a significant portion of the IgG antigen-binding site retains extensive secondary structure even after acidification (HAREN). Though there is overlap in this method, we believe this is a good indicator of the nature of neutralization. Measuring each of the components, we found that the HSEN and ASEN values were remarkably similar in males and females (Figure 1, B and C). There was a difference in the effect of age in HSEN and ASEN between the sexes. In males there was a decrease in HSEN (Figure 2C) and an increase in ASEN (Figure 2E) with age. There was no age component to any neutralization factor in females (Figure 2). Given that there is a significant difference in TEN between the sexes, but little difference in HSEN and ASEN, the HAREN portion of neutralization makes up this difference (Figure 1D). Acidification primarily affects the CH2 domain of the Fc fragment of immunoglobulins while the Fab fragment retains a significant fraction of secondary, and possibly, some tertiary structure [33,46]. We propose that this portion of endotoxin neutralization is mostly due to high-affinity immunoglobulins developed in response to exposure to Gram-negative bacteria that retain secondary structure after acidification and heat.

A precise etiology of IBDs has not been identified; rather it is a complex interaction of factors. Using family studies it has been established that there is a genetic component to IBD and some specific gene variants have been identified [47–51]. However, genetics only cause a propensity for the disease which is affected by other factors such as diet, smoking, birth control and emotional stress [52–55]. The most crucial factor in the development of IBD is most likely the makeup of bacterial flora in the intestines. Several studies have shown differences in the species and abundance of gut bacteria with some even demonstrating a correlation between the level of specific strains and disease severity [49,56–58]. IBDs are the result of excessive and inappropriate inflammation in the intestinal lining under specific environmental conditions in genetically predisposed people. Given that IBD involves chronic Gram-negative exposure, and currently has no dependable non-invasive test to monitor progression, we believe it is an attractive candidate to test the validity of endotoxin neutralization as a biomonitor.

In males with IBD the level of TEN was significantly elevated (Figure 3A). The difference was such that a line of demarcation could be established to differentiate the populations with over 95% accuracy. However, given that control females already have elevated TEN, most likely as a result of increased bacterial exposure, this metric is not a valuable indicator for this group. Therefore, measuring and comparing the individual components of endotoxin neutralization is necessary to develop a metric to measure all patient types. In both males and females we found a marked decrease in HSEN (Figure 4) with a corresponding increase in ASEN (Figure 5). This signifies the change from an innate immune response, dependent on heat-labile factors such as enzymes and IgM, to adaptive immunity as a response to chronic bacterial exposure, consistent with previous anti-endotoxin immunoglobulin studies (10). These individual components of neutralization provide a pattern of ENR that is consistent with the known mechanisms of CD and UC. ENR roughly doubles in patients with UC in both males and females (factoring in the omission of the outlier in the female UC group) (Figure 7). This increase is statistically significant and can demarcate control from UC patients with 77% accuracy. This doubling is a result of the decrease in HSEN and increase in ASEN. This is also the case with CD, but due to an even more elevated level of HAREN, the values are even higher. This increase is almost 9-fold in males and 5-fold in females. In keeping with our model, the higher level of HAREN in CD, as compared to control or UC, is the result of higher levels of intestinal permeability leading to a greater repertoire of anti-endotoxin IgG. This result is consistent with previous observations of intestinal permeability, circulating endotoxin and immunoglobulins in comparing UC and CD. Lending further credence is the observation of a strong correlation between ENR and disease severity and/or medication (Figure 8) [1,13,18]. With both males and females the highest ENRs were measured in patients that had severe forms of CD and/or were untreated. Thought this correlation lacks the sample size to have statistical significance, it fits with the overall findings of this report and therefore we include them as supporting data as we expand the scope of experiments in our laboratory.

The ultimate goal of this report was to develop a simple system to monitor bacterial exposure. The TEN values show great potential to do this in males and we hypothesize it may be valuable in cases of acute infection, such as sepsis. However, with chronic endotoxin exposure in all patients the ENR value, which describes the ratio of immune components responsible for long-term protection to those appearing quickly after infection, is more valuable. This report indicates that ENR is valuable in discerning IBD from control patients as well as having the potential to measure disease severity and the effectiveness of medications. The development of a functional biomonitor for IBD is of great importance since it currently has no dependable, non-invasive test. Imaging procedures, such as ultrasound, and serum tests, such as erythrocyte sedimentation rate and the level of C-reactive protein (CRP), are not reliable [59]. The most promising serum marker to date has been CRP. However, it is only a measure of existing inflammation and provides no information about disease history [60]. Many reports have also shown a tight correlation between anti-endotoxin immunoglobulins and IBD [1,13,16,18]. One method of measuring this specific subset of immunoglobulins is so sensitive that it is a predictive marker of endotoxin exposure even when circulating endotoxin is not detectable [10]. These results are possible because, instead of measuring total immunoglobulin, this method is specific for the immune response against endotoxin. Also, unlike CRP, it is not just a “snapshot” of the disease, but provides an historical context of the patient. We believe the method detailed in this report takes this approach one step further and measures only functionally active anti-endotoxin immunoglobulins as well as other endotoxin-specific mechanisms of the innate response to endotoxin.

## Supporting Information

Figure S1 **Endotoxin neutralization ratio by age.**
ENR was determined for each sample using the formula (ASEN + HAREN)/HSEN using the average of 3 replicates for each value. The ENR values were plotted against patient age and a linear regression line was fit to the data. The *r* value is indicated. The effect of age on ENR is much less than the individual components. (A) In males, ENR increases slightly with age (*r* = 0.2946). (B) In females there is minimal change (*r* = -0.1389).
(TIF)
