# Supplementary figures and images for: Endotoxin Neutralization as a Biomonitor for Inflammatory Bowel Disease
Source: PLoS One. 2013 Jun 24;8(6):e67736. doi: 10.1371/journal.pone.0067736 (PMC3691319; doi:10.1371/journal.pone.0067736)

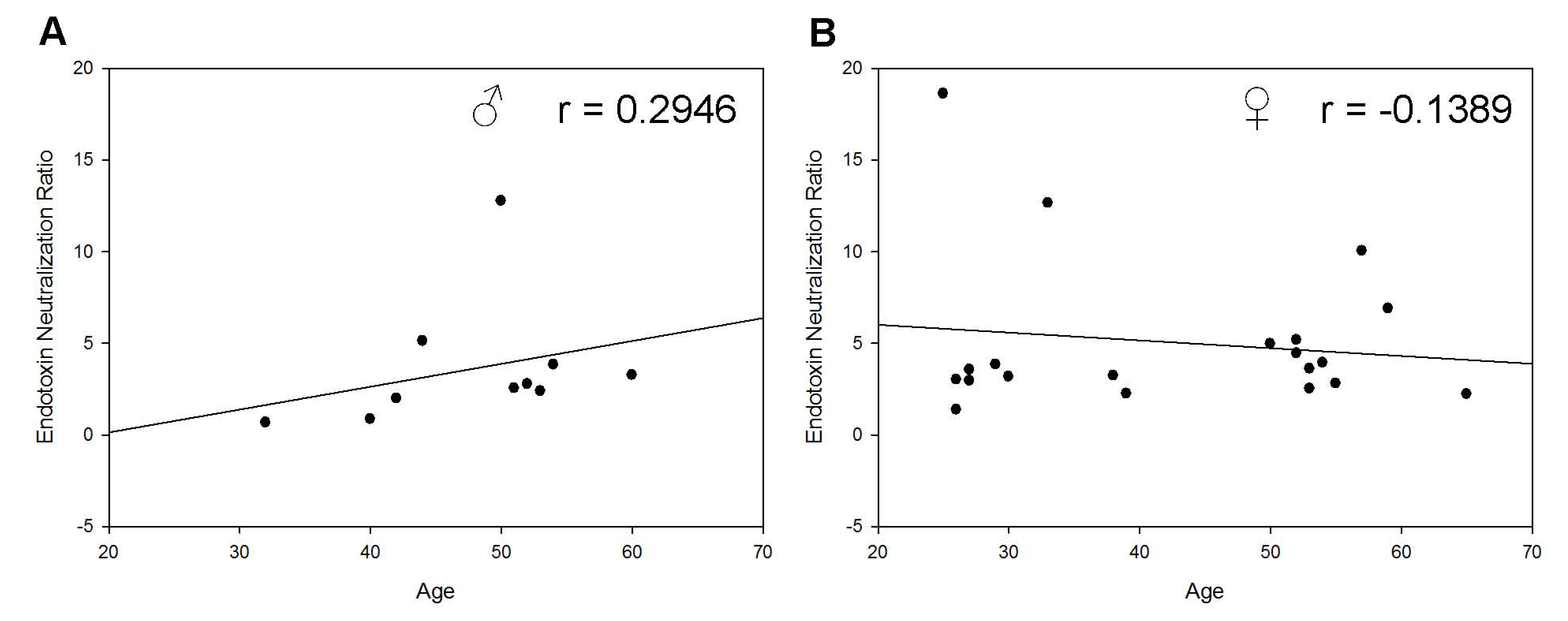

Supplement: Figure S1 — Endotoxin neutralization ratio by age. ENR was determined for each sample using the formula (ASEN + HAREN)/HSEN using the average of 3 replicates for each value. The ENR values were plotted against patient age and a linear regression line was fit to the data. The r value is indicated. The effect of age on ENR is much less than the individual components. (A) In males, ENR increases slightly with age (r = 0.2946). (B) In females there is minimal change (r = -0.1389). (TIF) [file pone.0067736.s001.tif]
